# Supplementary material for: Deep Learning-Based Synthetic CT for Personalized Treatment Modality Selection Between Proton and Photon Therapy in Thoracic Cancer
Source: Cancers (Basel). 2025 May 3;17(9):1553. doi: 10.3390/cancers17091553 (PMC12071890; doi:10.3390/cancers17091553)
Supplement: Supplementary file 1 [file cancers-17-01553-s001.zip › cancers-3574189-Supplementary.pdf]

Table S1 Planning parameters of VMAT and IMPT plans for sCT and pCT

Case	Treatment technique	Rx(Gy)/ Fx	Plan parameters		DVH constraints	
			Energy	Beam angle	Target	OAR
Case 1	VMAT	60/30	6X-FFF	10-179 CW	PTV	Lung conventional constraints
	PBS		50-250P	T270G20 T0G65	ITV	
Case 2	VMAT	60/30	6X-FFF	30-179 CW	PTV	
	PBS		10-250P	T270G185 T270G165 T0G0 T0G35	CTV	
Case 3	VMAT	50/20	6X	355-175 CW	PTV	
	PBS		10-250P	T270G180 T0G35 T0G0	ITV	
Case 4	VMAT	50/20	6X	0-181 CW	PTV	
	PBS		10-250P	T270G185 T270G155	ITV	
Case 5	VMAT	50/20	6X	179-181 CCW	PTV	
	PBS		50-250P	T270G185 T270G160 T180 G150	ITV	
Case 6	VMAT	60/28	6X-FFF	10-181CW	PTV	
	PBS		10-250P	T180G30 T0G20 T180G180	ITV	
Case 7	VMAT	54/30	6X	30-179 CW	PTV	
	PBS		50-250P	T270G155 T270G185	ITV	
Case 8	VMAT	60/30	6X	179-181 CCW	PTV	
	PBS		10-250P	T270 G30 T270G355	ITV	
Case 9	VMAT	60/30	6X	179-181CCW	PTV	
	PBS		50-250P	T270G185 T180G155 T0G150	ITV	
Case 10	VMAT	66/30	6X	30-181 CCW	PTV	
	PBS		50-250P	T0G45 T45G45 T0G355	ITV	
Case 11	VMAT	30/10	6X	179-181CCW	PTV	
	PBS		50-250P	T0G180 T0G155 T180G155	CTV	
Case 12	VMAT	50/5	6X-FFF	45-179 CW	PTV	Lung SBRT 5x constraints
	PBS		10-250P	T0G185 T270G155 T0G90	ITV	
Case 13	VMAT	50/5	6X	179-330 CCW	PTV	
	PBS		50-250P	T30G135 T0G185 T270G185	ITV	
Case 14	VMAT	48/4	6X-FFF	0-179 CW	PTV	Lung SBRT 4x constraints
	PBS		50-250P	T45G50 T0G355 T0G120	ITV	

Case 15	VMAT	48/4	6X-FFF	359-181 CW	PTV
	PBS		50-250P	T0G150 T180G95 T180G45	OTV

Rx: prescription. Fx: fraction. PBS: pencil beam scanning. SRS: stereotactic radiosurgery. SBRT: stereotactic body radiation therapy. T: couch angle. G: gantry angle. CW: clockwise. CCW: counterclockwise.

The detailed OAR constraints in Table S1 are shown below. The structure names for the target are bolded for proton plans and unbolded for photon plans. The OAR structure constraints are the same for both photon and proton plans.

Lung Conventional (Lung) Constraints					
Priority	Structure Template	Type	Prescription	Constraint	Goal
1	PtvLung/**ITV**	Target	High: 6000cGy	D99% ≥	93%
2	PtvLung/**ITV**	Target	High: 6000cGy	V100% ≥	95%
3	PtvLung/**ITV**	Target	High: 6000cGy	V110% ≤	1%
4	brachial_plex_l	OAR		Max ≤	6300cGy
5	brachial_plex_r	OAR		Max ≤	6300cGy
6	cord	OAR		Max ≤	5000cGy
7	cord_prv	OAR		Max ≤	5500cGy
8	cord_prv	OAR		V5500cGy ≤	0.1cc
9	esophagus	OAR		Mean ≤	3400cGy
10	esophagus	OAR		V3500cGy ≤	50%
11	esophagus	OAR		V5000cGy ≤	45%
12	esophagus	OAR		V5500cGy ≤	40%
13	esophagus	OAR		V6000cGy ≤	5%
14	heart	OAR		Max ≤	6200cGy
15	heart	OAR		Mean ≤	2000cGy
16	heart	OAR		V3000cGy ≤	46%
17	heart	OAR		V4000cGy ≤	33%
18	lung_total	OAR		Mean ≤	2000cGy
19	lung_total	OAR		V1000cGy ≤	42%
20	lung_total	OAR		V2000cGy ≤	35%

Lung SBRT 5Fx (Lung) Constraints					
Priority	Structure Template	Type	Prescription	Constraint	Goal
1	PtvLung/**ITV**	Target	High: 5000cGy	D99% ≥	90%
2	PtvLung/**ITV**	Target	High: 5000cGy	V100% ≥	95%
3	PtvLung/**OTV**	Target	High: 5000cGy	CI 100% ≤	1.2-1.5
4	PtvLung/**OTV**	Target	High: 5000cGy	CI 50% ≤	4.42-5.42
5	ItvLung	Target	High: 5000cGy	V110% ≥	20%

6	brachial_plex_l	OAR		Max ≤	3050cGy
7	brachial_plex_l	OAR		V2700cGy ≤	3cc
8	brachial_plex_r	OAR		Max ≤	3050cGy
9	brachial_plex_r	OAR		V2700cGy ≤	3cc
10	bronch_tree_nona	OAR		Max ≤	4000cGy
11	bronch_tree_nona	OAR		V1650cGy ≤	4cc
12	bronch_tree_prox	OAR		Max ≤	105%
13	bronch_tree_prox	OAR		V1800cGy ≤	4cc
14	chestwall	OAR		V3000cGy ≤	30cc
15	cord	OAR		Max ≤	3000cGy
16	cord	OAR		V2300cGy ≤	0.35cc
17	esophagus	OAR		Max ≤	105%
18	esophagus	OAR		V2750cGy ≤	5cc
19	esophagus_nonadj	OAR		Max ≤	3500cGy
20	esophagus_nonadj	OAR		V1950cGy ≤	5cc
21	great_vessels	OAR		Max ≤	105%
22	great_vessels	OAR		V4700cGy ≤	10cc
23	great_vessels_na	OAR		Max ≤	5300cGy
24	great_vessels_na	OAR		V4700cGy ≤	10cc
25	heart	OAR		Max ≤	105%
26	heart	OAR		V3200cGy ≤	15cc
27	heart_nonadj	OAR		Max ≤	3800cGy
28	heart_nonadj	OAR		V3200cGy ≤	15cc
29	liver	OAR		MVS2010cGy ≥	700cc
30	lung_total	OAR		V2000cGy ≤	10%
31	lung_total	OAR		MVS1210cGy ≥	1500cc
32	lung_total	OAR		MVS1300cGy ≥	1000cc
33	skin	OAR		Max ≤	3200cGy
34	skin	OAR		V3000cGy ≤	10cc
35	stomach	OAR		Max ≤	3200cGy
36	stomach	OAR		V1800cGy ≤	10cc
37	trachea	OAR		Max ≤	105%
38	trachea	OAR		V1800cGy ≤	4cc
39	trachea_nonadj	OAR		Max ≤	4000cGy
40	trachea_nonadj	OAR		V1650cGy ≤	4cc

Lung SBRT 4Fx (Lung) Constraints					
Priority	Structure Template	Type	Prescription	Constraint	Goal
1	PtvLung/**ITV**	Target	High: 4800cGy	D99% ≥	90%
2	PtvLung/**ITV**	Target	High: 4800cGy	V100% ≥	95%
3	PtvLung/**OTV**	Target	High: 4800cGy	CI 100% ≤	1.2-1.5
4	PtvLung/**OTV**	Target	High: 4800cGy	CI 50% ≤	4.627-5.69
5	ItvLung	Target	High: 4800cGy	V110% ≥	20%
6	brachial_plex_l	OAR		Max ≤	2720cGy
7	brachial_plex_l	OAR		V2370cGy ≤	3cc
8	brachial_plex_r	OAR		Max ≤	2720cGy

9	brachial_plex_r	OAR	V2370cGy ≤	3cc
10	bronch_tree_prox	OAR	Max ≤	3500cGy
11	bronch_tree_prox	OAR	V1580cGy ≤	4cc
12	chestwall	OAR	V3000cGy ≤	30cc
13	cord	OAR	Max ≤	2600cGy
14	cord	OAR	V2050cGy ≤	0.35cc
15	esophagus	OAR	Max ≤	2520cGy
16	esophagus	OAR	V1860cGy ≤	5cc
17	great_vessels	OAR	Max ≤	4900cGy
18	great_vessels	OAR	V4300cGy ≤	10cc
19	heart	OAR	Max ≤	3400cGy
20	heart	OAR	V2800cGy ≤	15cc
21	liver	OAR	MVS2010cGy ≥	700cc
22	lung_total	OAR	V2000cGy ≤	10%
23	lung_total	OAR	MVS1210cGy ≥	1500cc
24	lung_total	OAR	MVS1300cGy ≥	1000cc
25	skin	OAR	Max ≤	2800cGy
26	skin	OAR	V2600cGy ≤	10cc
27	stomach	OAR	Max ≤	2710cGy
28	stomach	OAR	V1730cGy ≤	10cc
29	trachea	OAR	V1580cGy ≤	4cc

Table S2 Parameters for NTCP and EUD calculation of three selected OARs

OAR	Acute endpoint	a	γ_{50}	TD_{50} (Gy)
Esophagus	Perforation	19	4	68
Lung	Pneumonitis	1	2	24.5
Heart	Pericarditis	3	3	50
